# Survey study on sentinel lymph node biopsy: indications and perceived value among small animal surgical specialists

**DOI:** 10.3389/fvets.2025.1591877

**Published:** 2025-08-06

**Authors:** Lavinia Elena Chiti, Ester Luconi, Alessandra Ubiali, Patrizia Boracchi, Damiano Stefanello, Mirja Christine Nolff

**Affiliations:** ^1^Clinic for Small Animals Surgery, Vetsuisse Faculty, University of Zurich, Zurich, Switzerland; ^2^Dipartimento di Scienze Biomediche per la Salute, Università degli Studi di Milano, Milan, Italy; ^3^Dipartimento di Medicina Veterinaria e Scienze Animali, Università degli Studi di Milano, Lodi, Italy; ^4^Dipartimento di Scienze Biomediche e Cliniche, Università degli Studi di Milano, Milan, Italy

**Keywords:** sentinel lymph node, survey study, dog, cancer, lymphadenectomy

## Abstract

**Introduction:**

Despite the growing adoption of sentinel lymph node biopsy (SLNB) for nodal staging in dogs, standardized guidelines defining its specific indications remain lacking. This survey study aimed to assess the indications for SLNB among small animal surgical specialists and evaluate its perceived value.

**Methods:**

An online survey was distributed through the European College of Veterinary Surgeons (ECVS), comprising five sections: clinical practice type, nodal staging, lymphadenectomy, mapping techniques, and the perceived value of SLNB. Descriptive statistics summarized responses, while cluster analysis explored associations between clinical practice type and SLNB recommendations.

**Results:**

Seventy-four surgeons participated, with 74% practicing in non-academic and 26% in academic institutions. The majority (80%) performed lymphadenectomy with histopathological evaluation for nodal staging, and 53% recommended it for all malignant tumors, irrespective of nodal size. Additionally, 80% preferred SLNB over regional lymphadenectomy, particularly for specific tumor types (mast cell tumors, melanomas, carcinomas) or anatomical locations (oral tumors). Indirect CT lymphography was the most commonly used preoperative mapping technique (59.4%), yet only 23% of respondents combined preoperative and intraoperative mapping techniques. While 63% considered SLNB a reliable diagnostic tool, cluster analysis indicated that the type of clinical practice (academic vs. non-academic) did not influence SLNB recommendations.

**Discussion:**

Despite a low response rate (8.3%), findings suggest that SLNB is widely performed by specialists across different practice settings, particularly for select tumor types. However, with 20% not performing SLNB and 37% uncertain about its reliability, these results highlight the need for standardized guidelines to define clear indications and improve consistency in clinical decision-making.

## Introduction

Nodal metastases are a well-established negative prognostic factor for several canine malignancies, making their accurate identification crucial for tumor staging and treatment planning (1–6). Histopathological examination of surgically excised lymph nodes is considered the most reliable method for detecting nodal metastases, as clinical assessment, cytological evaluation, and advanced diagnostic imaging have limited specificity and carry a risk of false negatives (7–9). Ideally, the sentinel lymph node (SLN)—the first node in the lymphatic chain that drains a primary tumor—should be sampled to provide an accurate assessment of the lymphatic basin (10–12).

Given these considerations, there has been growing interest in SLN mapping and biopsy (SLNB) for nodal staging of various canine malignancies (13–15). The majority of available studies on SLNB focus on dogs with mast cell tumors (MCT), primarily due to the relatively high prevalence of this tumor (16, 17), its propensity for lymphatic spread (9, 11, 18, 19), and the therapeutic significance of lymphadenectomy (1, 2, 20, 21). SLNB in MCT, guided by various mapping techniques, has demonstrated high nodal detection rates (91–95%). Additionally, findings indicate that in 28–63% of cases, the SLN differs from the clinically expected regional lymph node (RLN), highlighting the utility of SLNB for MCT staging (11, 22, 23).

For canine oral tumors, despite the prognostic significance of lymphatic dissemination, recommendations for nodal staging remain inconsistent (24–26). Several sentinel lymph node mapping techniques have been validated for oral tumors (3, 27–29), yet a recent survey found that only 33% of respondents routinely incorporated SLNB into their practice (24). SLNB has also been shown to be feasible for canine mammary tumors, and there is growing interest in its application for apocrine gland anal sac adenocarcinoma (AGASACA). However, data on its impact on staging, prognosis, and treatment for these tumor types remain limited (30–33).

Despite the potential advantages of SLNB for nodal staging in canine malignancies—along with the feasibility and reliability of various mapping techniques and the reportedly low surgical morbidity associated with the procedure—the existing literature does not provide clear guidance for veterinary surgeons on when to perform SLNB instead of RLN extirpation or no lymphadenectomy at all, particularly for tumors other than MCT. Consequently, a standardized approach to nodal staging in canine oncology remains largely absent. Given the current understanding of the SLN’s role in canine oncology, this survey study aims to investigate the cases in which specialized small animal surgeons perform SLNB, which mapping technique they use, and assess their perceived value of this procedure.

## Materials and methods

### Survey design

An online survey was developed to assess the most commonly used approaches for nodal staging of malignant tumors in dogs, the circumstances in which SLNB is performed instead of other staging methods, and the perceived value of SLNB. The survey was created using a web-based institutional platform^1^ and was distributed via the listserv to small animal diplomates and residents of the European College of Veterinary Surgeons (ECVS).

To ensure content validity, the questionnaire was reviewed by two experts in small animal surgery (one ECVS diplomate, MCN, and one full professor, DS) and two professional statisticians (PB, EL). It consisted of 23 multiple-choice questions divided into five sections: (A) demographic and workplace characteristics, (B) nodal staging practices, (C) lymphadenectomy indications, (D) lymph node mapping techniques, and (E) perceived value of SLNB. Some questions allowed multiple responses regarding lymph node staging and mapping techniques, and a free-text field was included if respondents selected “Other” as an answer option. Prior to dissemination, the questionnaire was completed by two additional small animal ECVS diplomates, one primarily practicing orthopedic surgery and one soft tissue surgery, by one small animal ECVS resident (1^st^ year) and by one PhD student, who were not part of the expert review group. Their input was used to identify any ambiguous or unclear items and to ensure the questionnaire was practically relevant and understandable to the target audience. This step served as an informal pilot phase. A full version of the survey is provided as Supplementary material.

Participation in the survey was voluntary, and responses were anonymous. A presentation letter outlining the study’s objectives accompanied the survey link. Respondents were allowed to submit their responses even if some questions were left unanswered. The initial survey invitation was sent on April 20, 2023, followed by three reminder emails at three-week intervals to non-responders.

### Statistical analysis

The response rate was calculated as the proportion of surgeons who completed the survey relative to the total number of recipients (small animal ECVS diplomates and residents). Response frequencies for each question were reported as percentages, including the proportion of “non-respondents” for specific questions, as this may indicate items that were perceived as unclear or outside the respondent’s expertise.

To identify patterns among respondents with similar perspectives on SLNB, a cluster analysis was performed. The clustering included clinically relevant variables: institution type, annual volume of oncologic surgeries, indications for lymphadenectomy, circumstances favoring SLNB over regional lymphadenectomy, and the perceived value of SLNB.

Both agglomerative and divisive hierarchical clustering methods were evaluated to determine the most meaningful response groupings. For agglomerative clustering, multiple linkage methods were considered, including complete linkage, single linkage, average linkage, centroid linkage, Ward’s minimum variance method, median linkage, and McQuitty’s method (34). The divisive hierarchical clustering algorithm (DIANA) was used as the divisive method (35). Gower’s distance metric (36), suitable for categorical data, was applied to both clustering approaches.

For agglomerative clustering, solutions ranging from two to seven clusters were assessed. Several clustering validity indices—including within-cluster sum of squares, average intra-cluster and inter-cluster distances, Wb ratio, Dunn2 index, and average silhouette width—were calculated to determine the optimal number of clusters based on intra-group cohesion and inter-group separation (37, 38). The final selection of the best clustering solution was based on the overall performance of these indices, as well as the distribution of subjects within each cluster. The same procedure was applied to the divisive method.

Once the optimal clustering solution was identified, the composition of each cluster was analyzed. Descriptive statistics were used to summarize response distributions within clusters, and a heatmap was generated to visualize these distributions. A partition tree was constructed to identify the most discriminative variables across clusters, facilitating a more concise interpretation of cluster structures.

Finally, the clustering results were reviewed by the clinicians who designed the questionnaire, and the most clinically relevant and informative solution was selected (39).

All statistical analyses were conducted using R statistical software.^2^

## Results

### Demographics and clinical practice

The survey was distributed to 893 surgeons, with 74 participants, yielding a response rate of 8.3%. Of these, 55 (74.3%) worked in non-academic referral practices, 18 (24.3%) in academic institutions, and one (1.4%) in a first-opinion practice. The annual number of surgical oncology procedures performed per institution exceeded 200 in 27 cases (36.5%), ranged between 100–200 and 50–100 in 20 cases (27%) each, and was below 50 in seven cases (9.5%).

Among the respondents, 53 (71.6%) were ECVS diplomates, including three ACVS fellows in surgical oncology (4%), and 20 (27%) were ECVS residents. One respondent did not specify their ECVS status (resident vs. diplomate). Most participants (*n* = 38; 51.4%) had over 10 years of experience in small animal surgery, while 21 (28.4%) had 5–10 years, and 15 (20%) had less than 5 years of experience. Only 13 (17.6%) reported that surgical oncology comprised more than 50% of their workload.

### Nodal staging

One participant did not complete this section. A total of 59 (79.7%) respondents routinely performed lymphadenectomy and histological examination of lymph nodes in tumor-bearing dogs. Clinical assessment of node size and consistency, cytology of the RLN, and diagnostic imaging (ultrasound, CT) were commonly used (*n* = 58; *n* = 64; *n* = 69, respectively) but rarely in isolation. Of the 14 surgeons who did not routinely perform lymphadenectomy and histopathology, 13 relied on ultrasound, CT, clinical assessment, or cytology for nodal staging. Most respondents decided whether to sample a node (cytologically or histologically) based on clinical size (*n* = 54; 72.9%) or tumor type (e.g., MCT, melanoma, squamous cell carcinoma) (*n* = 17; 31.5%).

### Lymphadenectomy

Two participants did not complete this section, and four provided only partial responses. Regarding indications for lymphadenectomy, 39 (52.7%) respondents performed it for all cytologically/histologically confirmed malignant tumors, while 29 (38.2%) restricted it to specific tumor types (e.g., MCT, oral tumors, carcinomas, mammary tumors) with or without lymphadenomegaly. Forty-six (62.2%) surgeons removed clinically enlarged nodes, but only nine (12.2%) performed lymphadenectomy exclusively in such cases. Four surgeons never performed lymphadenectomy concurrent with tumor excision; however, two of these also reported performing it for specific tumor types. This discrepancy was considered a potential inconsistency, and for cluster analysis, these two respondents were included in the group performing lymphadenectomy for specific tumor types.

Fifty-nine respondents (79.7%) indicated that they sometimes removed the SLN instead of the RLN: 22 (29.7%) did so for all cytologically/histologically confirmed malignant tumors, 31 (41.9%) only for specific tumor types or locations (*n* = 15, MCT; *n* = 10, oral tumors; *n* = 5, AGASACA; *n* = 2, mammary tumors), and six (8.1%) only if the primary tumor had additional negative prognostic factors. Forty-two participants (56.8%) performed SLNB even when the RLN was clinically enlarged or cytologically metastatic, while 54 (72.9%) also considered SLNB in cases of scars from previous tumor excisions (*n* = 20, only if prior excision was incomplete; *n* = 24, only if the primary tumor had negative prognostic factors). Forty-three (58.1%) surgeons recommended removing tier-2 lymph nodes in addition to SLN or RLN if the latter was metastatic (*n* = 32) or if they believed it might impact prognosis (*n* = 9). Only five (6.8%) respondents considered the term “SLNB” appropriate for SLN surgical resection, whereas 53 (71.6%) preferred “sentinel lymph node extirpation” or “sentinel lymphadenectomy.”

### Mapping techniques

Seventeen participants did not respond to this section, and two stated they did not perform SLNB. Among the 55 remaining respondents, 44 (59.4%) used indirect computed tomographic lymphography (ICTL), either alone or combined with an intraoperative technique [methylene blue (MB) or near-infrared lymphography (NIRF-L)]. Other techniques included MB alone (*n* = 6; 8.1%), contrast-enhanced ultrasound (CEUS) (*n* = 3; 4%), lymphoscintigraphy (*n* = 1; 1.4%), and indirect radiographic lymphography (*n* = 1; 1.4%). Seventeen (23%) respondents combined preoperative and intraoperative mapping techniques, while 29 (39.2%) used only preoperative and five (6.8%) only intraoperative techniques. Twenty-eight (37.8%) excised extra nodes not identified by mapping but directly visualized during surgery. In cases where the RLN and SLN did not correspond, 29 (39.2%) surgeons removed the RLN only if it was enlarged or cytologically positive, 14 (18.9%) always removed the RLN, and 11 (14.9%) removed only the SLN.

### Value of sentinel lymphadenectomy

Eighteen respondents did not answer this section. Forty-seven (63.5%) considered SLNB followed by histological examination a reliable method for detecting nodal metastases. Thirty-three (44.6%) believed SLNB affected staging, treatment recommendations, and outcomes, 21 (28.4%) stated it impacted staging and treatment but not outcome, and one believed it influenced only staging.

Regarding changes in SLNB recommendations over the past five years, 27 (36.5%) surgeons indicated they had not previously recommended SLNB but now do, 20 (27%) had consistently recommended it, eight (10.8%) had never recommended it and still do not, and one (1.4%) had stopped recommending it. Fifty-three (71.6%) respondents believed SLNB will become more routine in canine oncologic surgery within the next decade.

### Cluster analysis

**Figure 1:**
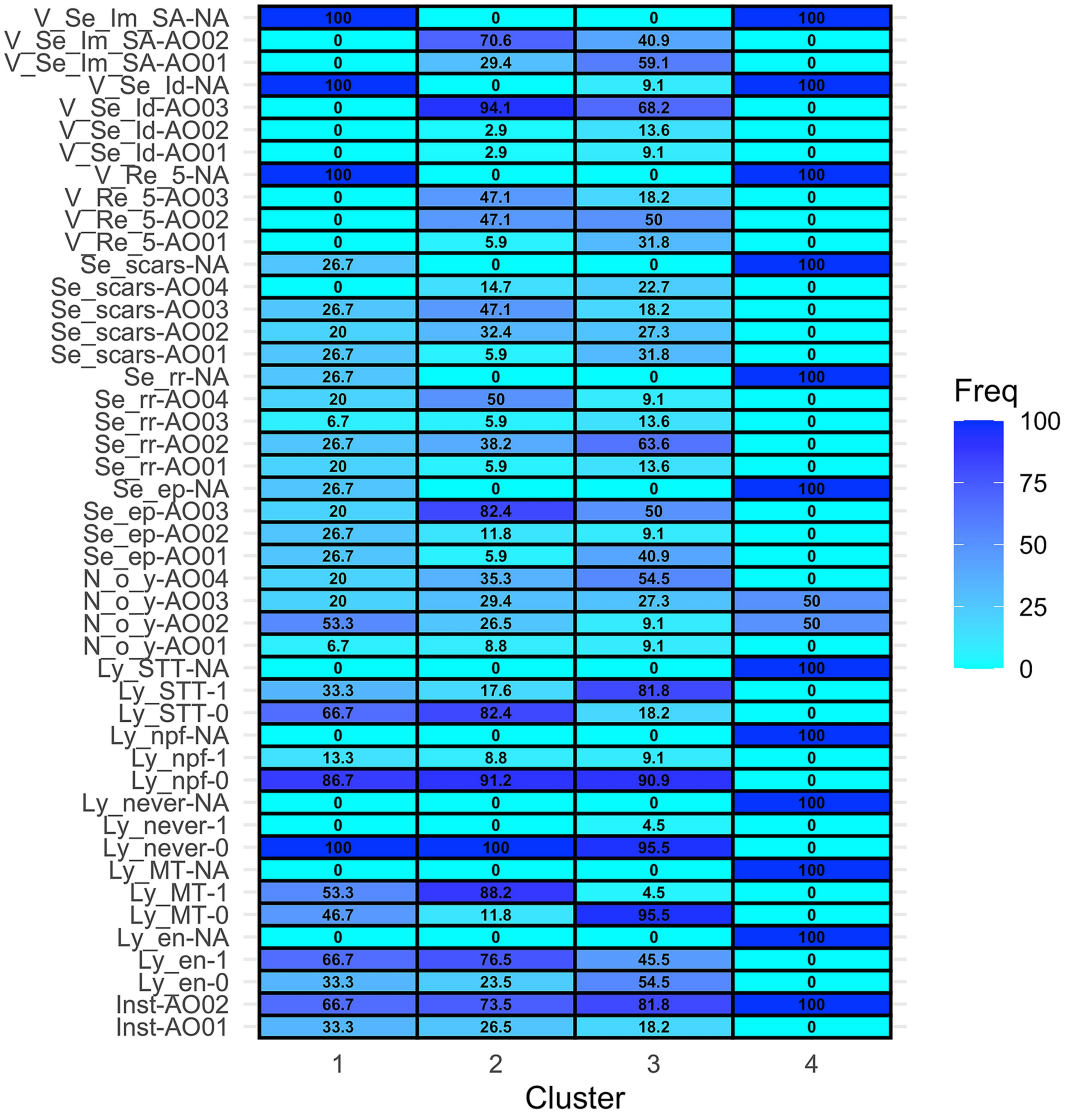
Heatmap representing the distribution of the answers (rows) in each cluster (columns). [Inst = Institution, Inst-AO01 = University teaching hospital, Inst-AO02 = Non-academic referral practice. Ly_en = I recommend lymphadenectomy concurrent with tumor excision if lymph nodes are clinically enlarged, Ly_en-0 = No, Ly_en-1 = Yes, Ly_en-NA = missing answer. Ly_MT = I recommend lymphadenectomy concurrent with tumor excision in case of Malignant Tumors, Ly_MT-0 = No, Ly_MT-1 = Yes, Ly_MT-NA = missing answer. Ly_never = Never I recommend lymphadenectomy concurrent with tumor excision, Ly_never-0: No, Ly_never-1 = Yes, Ly_never-NA = missing answer. Ly_npf = I recommend lymphadenectomy concurrent with tumor excision only if the primary tumor has negative prognostic factors, Ly_npf-0 = No, Ly_npf-1 = Yes, Ly_npf-NA = missing answer. Ly_STT = I recommend lymphadenectomy concurrent with tumor excision only for specific tumor types. Ly_STT-0 = No, Ly_STT-1 = Yes, Ly_STT-NA = missing answer. N_o_Y = How many surgical oncology procedures are performed in your institution every year? N_o_Y-Ao01 = <50, N_o_Y-Ao02 = 50–100, N_o_Y-Ao03 = 100–200, N_o_Y-Ao04 > 200, N_o_Y-NA = missing answer. Se_ep = I recommend resection of the sentinel lymph node(s) if the regional lymph node is clinically enlarged or cytologically positive for metastases. Se_ep- AO01 = No, Se_ep- AO02 = Only for specific tumor type or locations OR “Only if the primary tumor has negative prognostic factors,” Se_ep- AO03 = Yes. Se_ep-NA = missing answer. Se_rr = In which cases do you recommend sentinel lymphadenectomy rather than regional?, Se_rr -AO01 = Never, Se_rr -AO02 = Only for specific tumor types and locations, Se_rr -AO03 = Only if the primary tumor has negative prognostic factors, Se_rr -AO04 = In all cytologically/histologically confirmed malignant tumors, Se_rr -NA = missing answer. Se_scars = Do you suggest sentinel lymphadenectomy also in case of scars from previously excised tumors that did not have lymphadenectomy before? Se_scars- AO01 = No, never, Se_scars- AO02 = Only if previous excision was incomplete (R1 margins), Se_scars- AO03 = Only if the primary tumor has negative prognostic factors, Se_scars- AO04 = Yes, always. Se_scars- NA = missing answer. V_Re_5 = Have your recommendations for sentinel lymphadenectomy changed in the past 5 years? V_Re_5- AO01 = “I used NOT to recommend it and I am still NOT” OR “I used to recommend it, but now I do NOT recommend it anymore,” V_Re_5- AO02 = “I used NOT to recommend it, but now I am,” V_Re_5- AO03 = “I used to recommend it and I am still doing so,” V_Re_5-NA = missing answer. V_Se_Id = Do you believe that sentinel lymphadenectomy (followed by histological examination) is a reliable procedure to identify nodal metastases? V_Se_Id- AO01 = Only for specific tumor types or locations, V_Se_Id- AO02 = Depending on which mapping method is applied, V_Se_Id- AO03 = Yes, V_Se_Id-NA = “I do not have an opinion about it” OR missing answer. V_Se_Im_SA = What is your perceived impact of implementation of sentinel lymphadenectomy in small animal surgical oncology? V_Se_Im_SA-AO01 = “It does not have an impact on treatment choices and outcome of patients” OR “It affects staging” OR “It affects staging and treatment recommendations,” V_Se_Im_SA-AO02 = It affects staging, treatment recommendations and outcome V_Se_Im_SA-NA = missing answer].

Cluster analysis included 73 of 74 subjects, excluding the sole first-opinion practitioner. A divisive method identified four clusters of 15, 34, 22, and two subjects, respectively. The 4 clusters with characterization of the relative frequencies of each answer are summarized in the heatmap (Figure 1).

Clusters 1 and 2 included surgeons from both academic and non-academic referral practices, though non-academic referral surgeons were more prevalent in Cluster 2 (66.7% vs. 73.5%). Clusters 3 and 4 comprised only non-academic referral surgeons.

Cluster 1 had the highest proportion (53.3%) of surgeons performing 50–100 oncologic procedures per year, while Clusters 2 and 3 had more surgeons performing >200 procedures (35.3 and 54.5%, respectively). Cluster 4 was the one with the highest variability, including surgeons performing 50–200 procedures per year.

In Cluster 1, 100% of surgeons performed lymphadenectomy at least in some cases, with 66.7% doing so for clinically enlarged nodes and 53.3% for all malignant tumors. Regarding SLNB and mapping techniques, in this cluster there was a non-response rate of 26.7%. Twenty-seven % of surgeons in this group recommended SLNB instead of RLN removal selectively for specific tumor types or locations, while 20% recommended it for all confirmed malignant tumors, 7% only if the primary tumor had negative prognostic factors, and 20% never recommended SLNB instead of RLN removal. None of the respondents in cluster 1 answered to the questions relative to the perceived value of lymphadenectomy.

In Cluster 2, 100% of respondents performed lymphadenectomy concurrent with tumor excision, and 88.2% did so for all malignant tumors. Differently from cluster 1, most of the respondents in cluster 2 performed SLNB either for all malignant tumors (50%) or only for specific tumor types/locations (38.2%). Sentinel lymph node biopsy is performed by 82.4% of the surgeons in this cluster if the RLN is clinically enlarged or cytologically positive for metastases. In this cluster 47.1% of the surgeons used to suggest SLNB 5 years ago and they still do it, and another 47.1% has started recommending it lately. Ninety-four percent believe that SLNB is a reliable procedure and 70.6% declare that it has an impact on staging, treatment, and outcome.

In Cluster 3 lymphadenectomy is also performed by 100% of subjects, but mainly for specific tumor types (81.8%). In this cluster there is a predominance of surgeons that perform SLNB only for specific tumor types or locations (63.6%). In this cluster, 31.8% of the subjects do not currently perform SLNB, and another 50% have started performing it in the last years. Most (68.2%) of the subjects in this cluster believe that SLNB is a reliable procedure.

Cluster 4 contained two respondents who did not answer questions regarding lymphadenectomy or SLNB.

**Figure 2:**
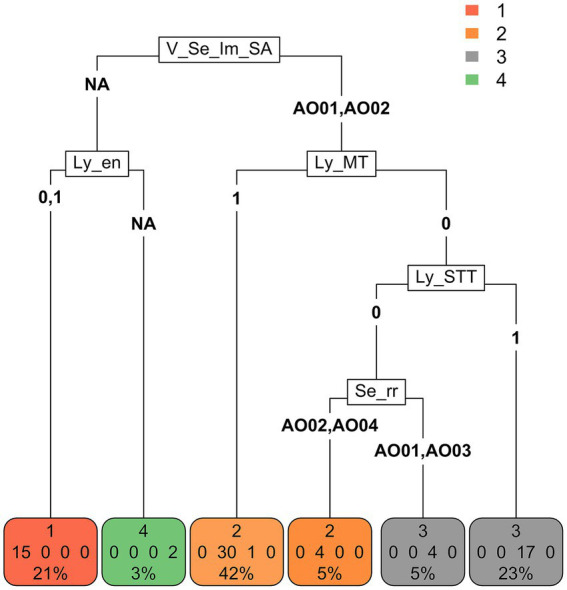
Partition tree. The variable that is represented first (at the top) (V_Se_lm_SA) is the one that discriminates clusters the most, followed by two variables (Ly_en and Ly_MT) (on the same level), which discriminate clusters asymmetrically with respect to the first and further separations. Finally, the procedure allocates the subjects into terminal nodes, which should correspond to the original classification of the clusters. The subjects were separated into 4 terminal nodes (bottom), which give the subject classifications provided by the partition tree procedure. As it is an approximate procedure, not all subjects were correctly classified according to the cluster they belong to, and classification errors are also reported. For example, in terminal node 1, there are no classification errors (all 15 subjects actually belong to cluster 1). In terminal node 2 (left) there are three classification errors: 30 subjects actually belong to cluster 2 and 1 to cluster 3. [V_Se_Im_SA = What is your perceived impact of implementation of sentinel lymphadenectomy in small animal surgical oncology? V_Se_Im_SA-AO01 = “It does not have an impact on treatment choices and outcome of patients” OR “It affects staging” OR “It affects staging and treatment recommendations,” V_Se_Im_SA-AO02 = It affects staging, treatment recommendations and outcome V_Se_Im_SA-NA = missing answer. Ly_en = I recommend lymphadenectomy concurrent with tumor excision if lymph nodes are clinically enlarged, Ly_en-0 = No, Ly_en-1 = Yes, Ly_en-NA = missing answer. Ly_MT = I recommend lymphadenectomy concurrent with tumor excision in case of Malignant Tumors, Ly_MT-0 = No, Ly_MT-1 = Yes. Ly_STT = I recommend lymphadenectomy concurrent with tumor excision only for specific tumor types. Ly_STT-0 = No, Ly_STT-1 = Yes. Se_rr = In which cases do you recommend sentinel lymphadenectomy rather than regional? Se_rr -AO01 = Never, Se_rr -AO02 = Only for specific tumor types and locations, Se_rr -AO03 = Only if the primary tumor has negative prognostic factors, Se_rr -AO04 = In all cytologically/histologically confirmed malignant tumors, Se_rr -NA = missing answer].

Partition tree analysis (Figure 2) identified the perceived impact of SLNB as the most discriminating variable for cluster partition, followed by lymphadenectomy criteria (removal of the nodes only if enlarged and removal of the nodes in all malignant tumors).

## Discussion

The high sensitivity of histopathology in detecting nodal metastases in excised lymph nodes has driven the adoption of lymphadenectomy for various canine malignancies, and, more recently, the potential discordance between the clinically expected RLN and the SLN, has led to an increased utilization of SLN mapping techniques (3, 11, 12, 22, 28, 29). However, these techniques are not universally available in small animal clinical practice, and tumor-specific guidelines for SLN biopsy (SLNB) remain undefined (24, 26). The heterogeneity of the available literature on SLNB underscores variations in institutional practices, including inconsistencies in mapping techniques, in the combination of preoperative and intraoperative techniques, and differences in SLNB protocols and outcome measures (11, 29, 40, 41). Establishing general guidelines for SLNB first requires a better understanding of current clinical indications and the perceived value of this procedure among small animal surgical specialists.

Based on the present survey, approximately 80% of respondents routinely perform either regional or sentinel lymphadenectomy for staging canine malignancies, with over 50% recommending it regardless of tumor type or RLN size. A previous survey on nodal staging in canine head and neck tumors reported that 41–80% of respondents opted for lymphadenectomy only in cases of clinical or cytological suspicion of metastases (24). This discrepancy may reflect an increasing interest in detecting subclinical nodal metastases at an earlier stage.

SLNB was preferred over RLN removal by 80% of respondents, at least in selected cases. However, considerable variability was observed in the specific indications for SLNB. Thirty percent of surgeons performed SLNB for all cytologically or histologically confirmed malignant tumors, while 42% limited its use to specific tumor types or anatomical locations, and 8% reserved it for cases with additional negative prognostic factors. This high degree of variability is unsurprising, given the current lack of strong evidence defining SLNB indications. The absence of standardized guidelines highlights the need for evidence-based recommendations to ensure a more uniform standard of care among specialists. Among surgeons performing SLNB only for specific tumor types or locations, the majority applied it to mast cell tumors (MCT) and oral tumors, likely reflecting the focus of most available literature on these malignancies (3, 23, 28, 29, 42, 43).

More than half of respondents (58.1%) recommended excision of second-echelon lymph nodes when metastases were detected in the RLN or SLN or when they believed that removing additional nodes could influence prognosis. In human oncology, it is well established that removal of second-tier lymph nodes in the presence of SLN metastases can reduce tumor burden and improve outcomes (44, 45). However, comparable evidence is lacking in dogs, and the prognostic benefits of removing second-tier nodes remain unassessed.

Interestingly, fewer than 10% of respondents considered the term “*sentinel lymph node biopsy*” an appropriate descriptor for sentinel lymph node resection, despite its widespread use in both veterinary and human literature. Standardizing definitions and terminology among specialists is crucial for ensuring consistency in scientific reporting and facilitating cross-study comparisons.

Regarding mapping techniques for SLNB, most respondents used indirect computed tomographic lymphography (ICTL) alone or in combination with methylene blue (MB) or near-infrared fluorescence lymphography (NIRF-L). The widespread use of ICTL is likely due to its accessibility and the number of studies supporting its application (12, 22, 28, 29, 33, 40, 43). However, ICTL detection rates vary widely (42–97%), and therefore combining it with intraoperative mapping techniques has been proposed to improve accuracy (12, 29). In one study, ICTL alone identified 42% of SLNs, whereas the detection rate increased to 100% when combined with intraoperative NIRF-L (29). In general, the combined use of preoperative and intraoperative mapping techniques or multiple intraoperative tracers has been shown to enhance SLN detection in both canine and human oncology (12, 29, 42, 46, 47). However, in this survey, only 23% of surgeons reported using a combined approach, while the majority relied on a single mapping technique (preoperative or intraoperative). This is concerning, as lower detection rates increase the risk of false negatives, potentially compromising the oncologic accuracy and clinical reliability of SLNB.

Regarding the perceived value of SLNB, most respondents considered it a reliable method for detecting nodal metastases and anticipated broader adoption in veterinary oncology over the next decade. This positive outlook further underscores the need for standardized recommendations to guide clinical implementation.

Cluster analysis revealed that institutional type (academic vs. non-academic) and annual surgical oncology caseload were not significant factors influencing recommendations or perceptions of SLNB. Although unexpected, this finding may be explained by the inclusion of board-certified surgical specialists, who may be more inclined to implement advanced procedures regardless of institutional affiliation. Given the low response rate, selection bias may have also influenced the results, as surgeons who chose to participate were likely those being more familiar and routinely performing lymphadenectomies, regardless of institutional type and caseload.

One of the clusters (Cluster 4) consisted of two respondents only who did not answer the questions related to SLNB. This cluster, although small and incomplete, was retained as it likely represents a subgroup of specialists who do not perform SLNB and excluding them would have introduced a selection bias. Hence, we chose not to merge or omit this cluster, while acknowledging its limited interpretability.

The most discriminating variable in the clustering process was the perceived impact of SLNB on oncologic management. Given the current lack of evidence-based guidelines—particularly for tumors other than MCT—it is reasonable that specialists base their decisions on the potential impact of SLNB on patient outcomes.

Another factor that may influence the decision to perform or not lymphadenectomy, either regional or sentinel, is the anatomical location of the lymph nodes, specifically if they are intracavitary or peripheral. The potential morbidity associated with intracavitary node dissection may indeed be perceived as unjustified in the absence of clear prognostic benefit. Although this variable was not directly included in our survey due to its explorative design, it was indirectly addressed through questions related to specific tumor types and locations, which often imply distinct nodal drainage pathways. Moreover, it is reasonable to assume that, with the increasing adoption of minimally invasive techniques for SLNB of intraabdominal and intrathoracic nodes (48), an intracavitary location may become less of a limitation to the procedure in the future.

The primary limitation of this study is the low response rate, which may introduce selection bias if non-respondents have significantly different characteristics from those who participated. However, the fact that European College of Veterinary Surgeons (ECVS) residents and diplomates represent a relatively homogeneous population, may have mitigated this source of bias. Additionally, non-response bias remains a concern, as surgeons who do not perform SLNB may have been less inclined to participate, potentially skewing results toward those who view SLNB favorably. A previous survey on cervical lymph node staging in canine oral tumors received responses from 87 clinicians; although the response rate was not reported, it was likely similar to that of the present study (24). This suggests a broader trend of low response rates in veterinary clinician surveys, possibly due to time constraints or lack of interest in survey-based research. Nonetheless, similar response rates have been reported in human surgical, medical, and radiation oncology surveys, and recent analyses suggest that this does not introduce significant bias (49–51).

Lastly, it would have been valuable to distribute the survey to medical oncologists to compare perspectives on lymphadenectomy and SLNB between surgical and medical specialists. However, relevant professional associations declined to disseminate the survey, preventing such a comparison.

In conclusion, while SLNB is recognized as a useful and reliable procedure, significant variability exists in its indications, including which tumor types necessitate the procedure and the selection of appropriate mapping techniques. The development of standardized, evidence-based guidelines is crucial for generating robust scientific data on the prognostic benefits of SLNB and, ultimately, for enhancing the standard of care for tumor-bearing dogs. Moreover, the broader adoption and dissemination of the technologies required for sentinel lymph node mapping in the coming years may improve surgeons’ sensitivity to this procedure and likely lead to better response rates in future studies.

## Data Availability

The raw data supporting the conclusions of this article will be made available by the authors, without undue reservation.

## Footnotes

^1^ https://www.limesurvey.org

^2^version 4.0.4, https://www.R-project.org.
