# Supplementary material for: Survey study on sentinel lymph node biopsy: indications and perceived value among small animal surgical specialists
Source: Front Vet Sci. 2025 Aug 6;12:1591877. doi: 10.3389/fvets.2025.1591877 (PMC12365617; doi:10.3389/fvets.2025.1591877)
Supplement: Supplementary file 1 [file Data_Sheet_1.docx]

**QUESTIONNAIRE ON SENTINEL LYMPH NODE BIOPSY IN SMALL ANIMAL**

1. **Type of clinical practice**
2. In which type of institution to you work?

- University teaching hospital
- Non-academic referral practice
- First-opinion practice

1. What is your degree of qualification?

- ECVS / ACVS Resident
- ECVS / ACVS diplomate
- ACVS fellow in surgical oncology
- PhD or other academic qualification
- National surgical specialization
- Other (please specify)

1. How many years of experience as a surgeon do you have?

- I do not perform surgery.
- 0-5
- 5-10
- >10

1. How many surgical oncology procedures are performed in your institution every year?

- <50
- 50-100
- 100 -200
- >200

1. How much of your work is dedicated to surgical oncology?

- 0-25%
- 25-50%
- 50%-75%
- 75%-100%

1. **Nodal staging**
2. Which modality do you use for nodal staging in cancer-patients? (multiple answers possible)

- Clinical assessment of size and consistency of the regional lymph nodes
- Cytology of the regional lymph node
- Diagnostic imaging (Ultrasound, CT)
- Lymphadenectomy and histological examination of the excised lymph nodes
- Others (please specify)

1. In which cases do you recommend further diagnostic for nodal staging? (Multiple answers are possible)

- If lymph nodes are clinically enlarged
- In all cytologically / histologically confirmed malignant tumors
- Only for specific tumor types (please specify)
- Never

1. **Lymphadenectomy**
2. In which cases do you recommend lymphadenectomy concurrent with tumor excision? (Multiple answers are possible)

- If lymph nodes are clinically enlarged
- In all cytologically / histologically confirmed malignant tumors
- Only for specific tumor types (please specify)
- Only if the primary tumor has negative prognostic factors
- Never

1. How many lymphadenectomies are performed at your institution yearly?

- <50
- 50-100
- 100 -200
- >200

1. In your opinion, is the term “sentinel lymph node biopsy” adequate to describe the surgical resection of the first lymph node(s) that drain a primary tumor?

- Yes
- No
- I don’t have an opinion about it
- I prefer “sentinel lymph node extirpation / resection / dissection
- I prefer “sentinel lymphadenectomy”

1. How often do you perform sentinel lymphadenectomy rather than regional lymphadenectomy?

- Never
- 0-25% of cases
- 25-50% of cases
- 50-75% of cases
- 75-100% of cases

1. In which cases do you recommend sentinel lymphadenectomy rather than regional?

- In all cytologically / histologically confirmed malignant tumors
- Only for specific tumor types and locations (please specify)
- Only if the primary tumor has negative prognostic factors
- Never

1. If the regional lymph node is clinically enlarged or cytologically positive for metastases, do you recommend resection of the sentinel lymph node(s)?

- Yes
- No
- Only for specific tumor types or locations (please specify)
- Only if the primary tumor has negative prognostic factors

1. Do you suggest sentinel lymphadenectomy also in case of scars from previously excised tumors?

- Yes, always
- No, never
- Only if previous excision was incomplete (R1 margins)
- Only if the primary tumor had negative prognostic factors

1. Would you consider resecting lymph nodes that belong to the next lymphocenter in the draining chain (Tier 2 lymph nodes), on top of SLN or RLN removal?
   - Yes, always
   - No, never
   - Only if RLN or SLN are positive for metastases
   - Only in specific cases (please specify)
2. **Mapping techniques**
3. Which mapping technique do you use more often for sentinel lymph node mapping?

- Methylene blue alone
- Indirect CT lymphography
- Near infrared lymphography
- Lymphoscintigraphy with or without methylene blue
- Contrast enhanced ultrasonography
- Other (please specify)

1. Do you usually combine more than one technique?

- Yes, I use multiple preoperative techniques
- Yes, I use multiple intraoperative techniques
- Yes, I combine a preoperative and intraoperative technique
- No, I use just one preoperative technique
- No, I use just one intraoperative technique

1. How many lymph nodes do you usually remove within the same sentinel lymphocenter?

- All the lymph nodes that I can identify intraoperatively, regardless of intraoperative mapping technique
- Only the lymph nodes that are identified intraoperatively with the mapping technique
- Only the first lymph node that is identified intraoperatively visually or with the intraoperative technique

1. In case of non-correspondence between the sentinel and regional lymph nodes, what do you usually recommend?

- Excision of the regional lymph node only
- Excision of the sentinel lymph node only
- Excision of both the sentinel and regional lymph nodes
- Excision of the sentinel lymph node, and of the regional only if enlarged or cytologically positive for metastases

1. **Value of sentinel lymphadenectomy**
2. Do you believe that sentinel lymphadenectomy (followed by histological examination) is a reliable procedure to identify nodal metastases?

- Yes
- No
- Only for specific tumor types or locations (please specify)
- Depending on which mapping method is applied
- I don’t have an opinion about it

1. What is your perceived impact of implementation of sentinel lymphadenectomy in small animal surgical oncology?

- It does not have an impact on treatment choices and outcome of patients
- It affects staging
- It affects staging and treatment recommendations
- It affects staging, treatment recommendations and outcome

1. Have your recommendations for sentinel lymphadenectomy changed in the past 5 years?

- I used NOT to recommend it and I am still NOT
- I used NOT to recommend it, but now I am.
- I used to recommend it and I am still doing so
- I used to recommend it, but now I do NOT recommend it anymore.

1. How do you believe that the recommendations for sentinel lymphadenectmy in pet animals would chance in 10-years’ time?

- Sentinel lymphadenectomy will be offered more often
- Sentinel lymphadenectomy will be offered less often
- The actual situation will remain unchanged
